# Irritable bowel syndrome in the community: a cross-sectional study looking at patient experiences and challenges with the low FODMAP diet

**DOI:** 10.3389/fgstr.2026.1930300

**Published:** 2026-10-23

**Authors:** Jared Fehlman, Westley Peng, Christian Pfenning, Hayley McCorkle, Judy W. Nee, William D. Chey, Ceciel Rooker, Sarah Ballou, Allen A. Lee, Prashant Singh

**Affiliations:** 1 Department of Medicine, Huntington Health an Affiliate of Cedars-Sinai, Pasadena, CA, United States; 2 Michigan Medicine, University of Michigan, Ann Arbor, MI, United States; 3 International Foundation for Gastrointestinal Disorders, Mount Pleasant, SC, United States; 4 Division of Gastroenterology, Department of Internal Medicine, Beth Israel Deaconess Medical Center, Boston, MA, United States; 5 Division of Gastroenterology and Hepatology, Department of Medicine, Michigan Medicine, Ann Arbor, MI, United States

**Keywords:** diet, functional bowel disease, irritable bowel syndrome, low FODMAP, nutrition

## Abstract

While a diet low in fermentable oligosaccharides, disaccharides, monosaccharides and polyols (FODMAP) is the most evidence-based dietary therapy for irritable bowel syndrome (IBS), data on patient experiences with the low FODMAP diet (LFD) remains sparse. Even less information exists examining patients’ experiences with the re-introduction and personalization phases of LFD, especially in the community setting. This cross-sectional study surveyed 387 participants from the mailing list of the International Foundation for Functional Gastrointestinal Disorders (IFFGD) about their experiences with LFD. Additionally, focus groups collected qualitative data about participants’ experiences with the LFD diet. Despite 70% of participants reporting perceived improvement of symptoms with FODMAP restriction, 70% of participants reported that LFD was either somewhat or extremely difficult to follow and 59% of participants reported continuing the restriction phase beyond 8 weeks. 41% of participants found the re-introduction phase somewhat or extremely difficult and two-thirds took >1 month to complete it. Only 30% and 25% of participants worked with a dietitian for restriction and re-introduction phases, with many receiving their information online or via app. Finally, over 80% of participants reported they would be interested in a simplified version of the LFD. While the LFD led to perceived symptom improvement in most IBS patients, it is associated with limitations, including its complexity and restrictiveness. Furthermore, food-related fear and anxiety, particularly during restriction and reintroduction phases, pose significant challenges. These findings underscore the practical limitations LFD has, and participants expressed a strong interest in a simpler, more convenient alternative.

## Introduction

Irritable bowel syndrome (IBS) affects approximately 4-11% of the global population (1–3). It imposes a substantial economic burden, both in direct medical costs and indirect costs from work presenteeism and absenteeism (4–8). Up to 84% of those with IBS report food-related symptoms, and therefore, it is not surprising that dietary therapies remain a common and popular treatment strategy for managing symptoms (9). A diet low in fermentable oligosaccharides, disaccharides, monosaccharides, and polyols (FODMAPs) is the most evidence-based dietary therapy for IBS and is superior to other dietary therapies in improving IBS symptoms (10–13). As a result, the low FODMAP diet (LFD) remains the most common dietary intervention recommended for IBS by gastroenterologists and dietitians/nutritionists.

Currently, the LFD is implemented as a 3-step process involving an initial 2–6-week restriction phase (Phase-1), followed by a rechallenge phase (Phase-2) to identify food triggers, and finally, a long-term personalization phase based on the outcome of the rechallenges (Phase-3). Thus, LFD is a complicated and time-consuming process with adherence rates ranging from 33-61% for the restriction phase alone (14). However, most of these studies were from tertiary care settings, administered by expert GI dietitians/nutritionists, did not focus on all three stages of the LFD, and did not address other key aspects of the diet outside adherence (e.g., ease of following, social impact etc.). There is scarce data on real-world patient experience with the LFD, especially outside tertiary care centers where most IBS cases are managed, and the availability of dietitians/nutritionists is much more limited. In this study, we aimed to evaluate real-world patient experiences in a community setting with all three stages of the LFD.

## Materials and methods

The study was approved by the Institutional Review Board at the University of Michigan (HUM00269351).

### Study design

The design for this study was an explanatory sequential design using both quantitative as well as qualitative approaches. Inclusion criteria for this study were adults age 18 years or older, the presence of a diagnosis of IBS, and previously attempted LFD. The length of time spent on the LFD did not play a part in affecting inclusion. Exclusion criteria included patients under the age of 18, and patients who had never participated in an LFD. The quantitative data collection occurred initially, followed by the qualitative data obtained from focus groups. Integration of the data was done when both quantitative and qualitative were analyzed simultaneously.

### Survey

Participants for the survey were individuals on the mailing list for the International Foundation for Gastrointestinal Disorders (IFFGD). Baseline characteristics of individuals included in the IFFGD mailing list are shown in **Supplementary Table 1**. The survey was sent out to all individuals on the mailing list in the form of email. The survey itself was composed of 17 total questions (**Supplementary Table 1**) and administered in English language. These questions were developed in conjunction with the IFFGD and were designed to specifically query participant’s experiences with all three components of the LFD: restriction, re-introduction and maintenance/personalization. Questions were newly created and not adapted from prior studies. Ineligible survey responses were not included in analysis. Missing questions were omitted from analysis. Questions assessed the participants’ experience with various LFD phases using a 5-to-7 point Likert scale and assessed barriers and facilitators to adherence with LFD. Survey data was collected from July 31^st^, 2024, to December 24^th^, 2025.

### Participants for the focused groups

To better understand patient’s experiences with the LFD at a deeper level, 8 participants were randomly selected from the survey group for qualitative focus groups designed to inquire about their experiences with the LFD. Given that these participants were chosen at random, no sampling or recruitment procedures were utilized for this portion of the study. Two separate focus groups were conducted virtually with 5 study participants in one group, and 3 participants in the other. Focus groups were led by a representative from IFFGD (CR) as well as two researchers (PS, AL). A focus group guide/template was generated by PS and AL in conjunction with IFFGD. Each focus group took approximately 45–60 minutes and included semi-structured questions about the participants’ feelings, thoughts, and behaviors during the three phases of the LFD, benefits and challenges to the diet, and any changes that they would like to see with the LFD. All participants provided verbal consent to be audio recorded. Audio recordings were transcribed by members of the research team (JF, WP, CP). The recordings were anonymized, and no personal information was included in the transcribed audio files.

### Survey data and qualitative analysis

Survey responses were downloaded and imported into R (v4.5.0) (15). Data were summarized using descriptive statistics. Categorical variables were compared using Fisher’s exact test. A two-tailed *P*-value <.05 was considered statistically significant. Qualitative data was analyzed using an iterative immersion/crystallization approach (16). Three authors (JF, WP, CF) independently read the focus group transcripts and identified major themes, which were then refined through a series of group discussions with the entire team. We have organized the results in this paper according to the most prominent themes that emerged in our analysis.

## Results

A total of 387 participants responded to the survey. Sixty-seven of these participants were excluded due to lack of clinician diagnosed IBS, leaving 320 respondents (82.7%) included for further analysis. Of these 320 participants, a final two hundred sixty-one participants (81%) reported trying the LFD and were included for further analysis. Of those who had not tried the LFD, when asked why, 25% reported the diet was too difficult to follow, 23% reported the diet was too restrictive to follow, 11% reported the diet was too expensive to follow, and 4% subjectively reported that they were unfamiliar with LFD/unaware it existed.

### Experience with restriction phase

When participants who had tried LFD were asked whether their symptoms improved with the restriction phase, 71% reported an improvement in symptoms, while the remaining participants reported no improvement. When asked who helped them navigate the restriction/elimination phase of the diet, only 30% replied they had the help of a dietitian/nutritionist. About half (48%) of participants utilized online resources for guidance, 31% used the Monash or other apps, 10% used a physician-provided handout, and 8% selected “other”. In total, 42% reported navigating the restriction/elimination phase on their own.

Most participants reported that the restriction phase was either extremely difficult (25%) or somewhat difficult to follow (45%, **Figure 1A**). Over two-thirds of participants also reported that this phase of the diet was either moderately (38%) or extremely (28%) restrictive (**Figure 1B**). When it came to satisfaction with the restriction/elimination portion of the diet, over 50% of respondents reported being either extremely or somewhat satisfied, where 30% of patients reported either being somewhat or extremely dissatisfied (**Figure 1C**). The duration participants followed the restriction phase varied and can be seen in **Figure 1D**. Forty-one percent of participants reported LFD restriction phase to be somewhat to extremely more expensive than their habitual diet (**Figure 1E**).

**Figure 1:**
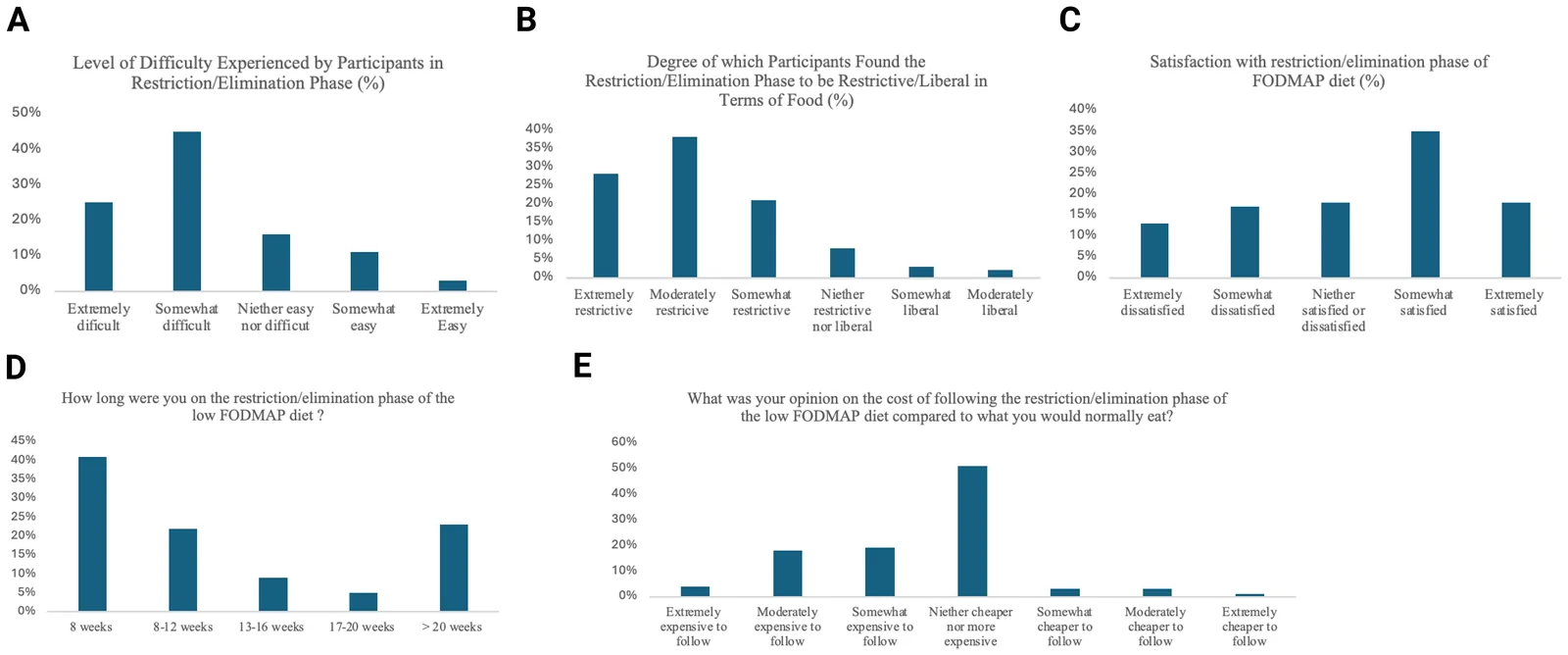
Patient experience in the restrictive phase of the LFD. Participant experience with restriction phase, including **(A)** The level of difficulty experienced by participants; **(B)** The degree of which participants found the restriction/elimination phase to be restrictive vs. liberal; **(C)** Satisfaction with restriction phase; **(D)** Length of restriction/elimination phase; and **(E)** Cost of the restriction phase of the LFD compared to normal.

### Experience with the reintroduction phase

When asked “Who helped you with the FODMAP re-introduction phase?”, half of respondents (50%) reported using online educational materials, while only 25% worked with a dietitian/nutritionist during reintroduction, and 17% reported being entirely unaware of the reintroduction phase (**Figure 2A**). The time taken to complete the reintroduction phase of the LFD varied greatly, with 33% reporting completing this phase in 8 weeks or less, 25% reporting 8–12 weeks, 15% reporting 13–16 weeks, 6% reporting 17–20 weeks and over 20% reporting more than 20 weeks (**Figure 2B**). Finally, 41% of participants reported the re-introduction phase as extremely or somewhat difficult (**Figure 2C**).

**Figure 2:**
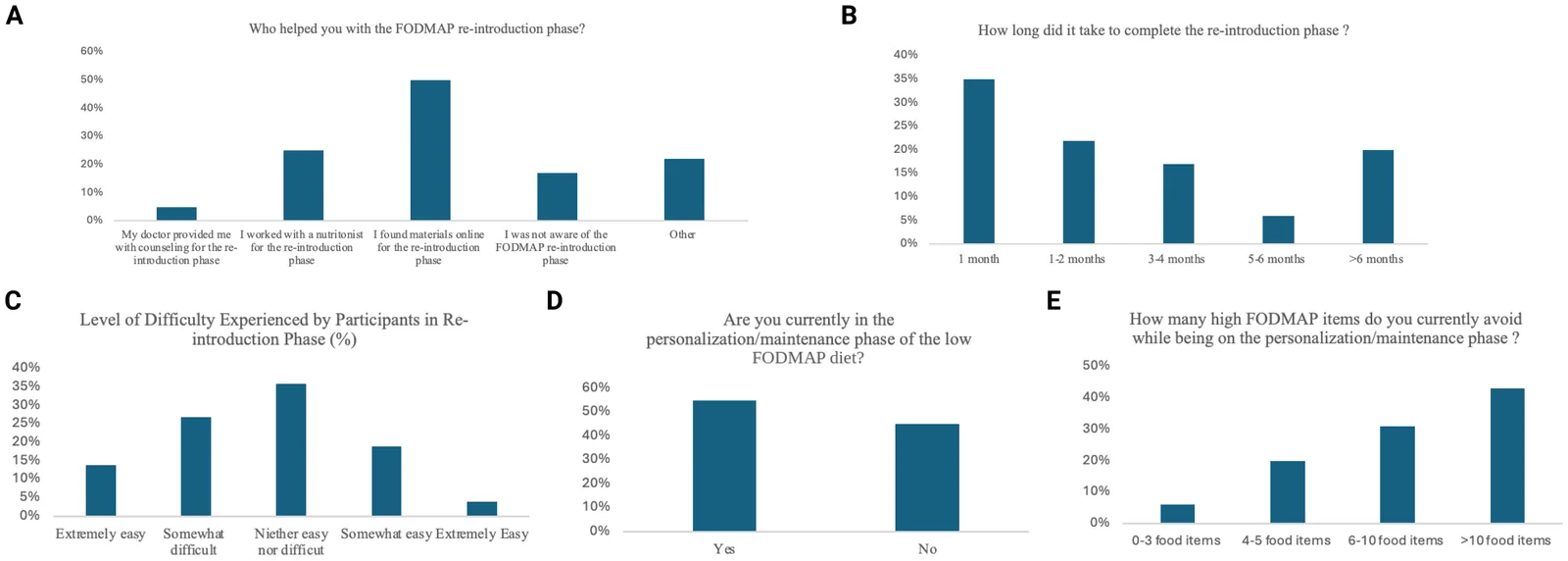
Participant experience in the re-introduction and personalization phase of the LFD. Participant experience with re-introduction and personalization phase, including **(A)** Who helped them navigate the re-introduction phase; **(B)** The length it took to complete the re-introduction phase; **(C)** The level of difficulty experienced by participants in the re-introduction phase; **(D)** Whether or not participants were in the personalization/maintenance phase; **(E)** How many high FODMAP foods participants continued to avoid.

### Experience with the personalization phase

Among those who responded to LFD restriction phase, only 55% reported being in the personalization phase at the time of completing the survey (**Figure 2D**). Among those who were in the personalization phase, 74% reported avoiding more than 5 food items (**Figure 2E**).

### Interest in simplified approach to LFD

Finally, participants were asked if they would be interested in a simplified version of the LFD, to which 83% of participants reported “yes”. While there is very limited evidence on utilizing a simplified LFD, a two-tiered approach has been suggested, consisting of a less restrictive first elimination phase followed by the full and more restrictive LFD for patients who still demonstrate refractory symptoms.

### Comparing findings in participants who used vs did not use a dietitian/nutritionist

In those who worked with a dietitian/nutritionist (39) there was a trend toward being more likely to finish all three phases of the LFD compared to those who did not work with a dietitian/nutritionist (62% vs. 46%, P > 0.05). Only 17.1% of participants who received counseling from a dietitian/nutritionist were in the restriction phase for more than 20 weeks compared to 26.7% who did not work with a dietitian/nutritionist (although this was not statistically significant, P > 0.05) (**Figure 3**). The full list of differences experienced by patients who did vs did not use a dietician can be seen in **Table 1**.

**Figure 3:**
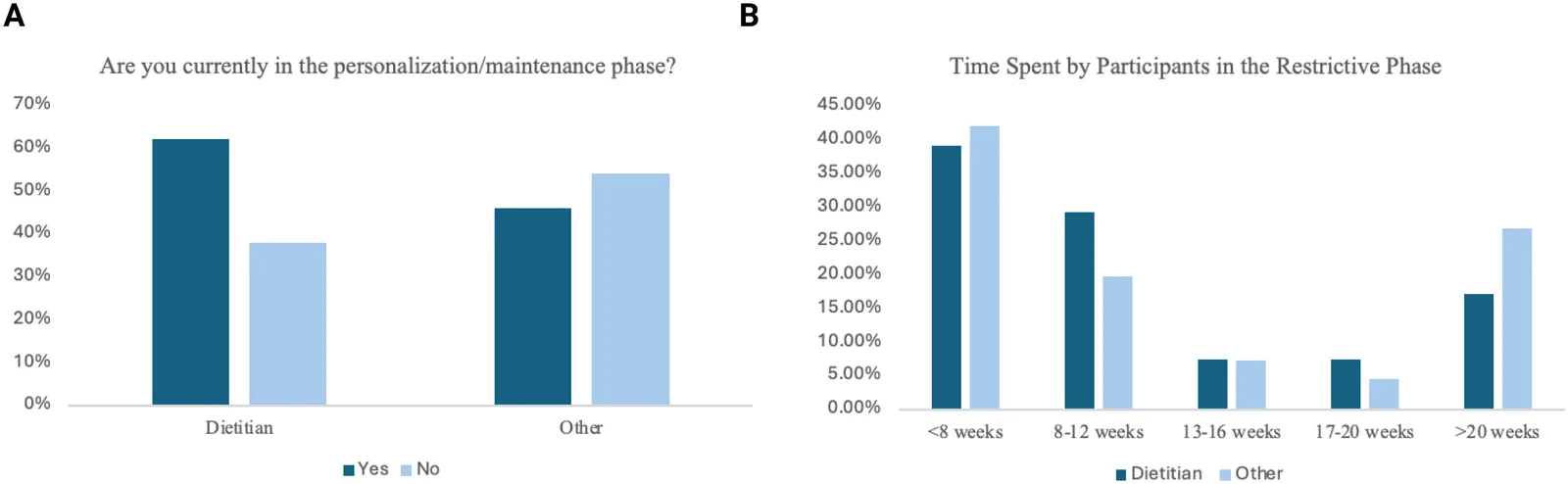
Participants who implemented LFD with and without the help of a dietician. Participant experience who navigated the LFD both with and without a dietitian. **(A)** Percentage of participants who were currently in the personalization/maintenance phase; **(B)** The time spent by participants in the restrictive phase of the LFD.

**Table 1:** Patients who implemented LFD with and without the help of a dietitian.

a: Patient experiences with restriction/elimination phase of LFD with and without a dietitian			
Difficulty of restriction phase			
Response	Dietitian	Other	P-values
Extremely difficult	21.95%	30.36%	0.417
Somewhat difficult	58.54%	40.18%	0.046
Neither easy nor difficult	12.20%	14.29%	1.000
Somewhat easy	2.44%	10.71%	0.187
Extremely easy	4.88%	4.46%	1.000
Time spent in restriction phase			
<8 weeks	39.02%	41.96%	0.853
8-12 weeks	29.27%	19.64%	0.272
13-16 weeks	7.32%	7.14%	1.000
17-20 weeks	7.32%	4.46%	0.442
>20 weeks	17.07%	26.79%	0.287
Level of restriction/liberalization patients found the restrictive phase			
Extremely Restrictive	28.21%	24.32%	0.671
Moderately Restrictive	46.15%	42.34%	0.710
Somewhat Restrictive	17.95%	20.72%	0.818
Neither Restrictive nor liberal	7.69%	9.01%	1.000
Somewhat liberal	0.00%	1.80%	1.000
Moderately liberal	0.00%	1.80%	1.000
Cost associated with restriction phase			
Moderately cheaper to follow	0.00%	0.00%	–
Neither cheaper nor more expensive	59.00%	47.75%	0.266
Somewhat expensive to follow	20.50%	22.52%	1.000
Moderately expensive to follow	18.00%	25.23%	0.390
Extremely expensive to follow	2.50%	4.50%	1.000
Satisfaction with restriction phase of LFD			
Extremely dissatisfied	17.95%	14.29%	0.609
Somewhat dissatisfied	20.51%	16.07%	0.623
Neither satisfied nor dissatisfied	0.00%	21.43%	0.0006
Somewhat satisfied	33.33%	32.14%	1.000
Extremely satisfied	28.21%	16.07%	0.105
b: Patient experiences with re-introduction and personalization phase of LFD with and without a dietitian			
Difficulty in re-introduction phase			
Response	Dietitian	Other	P-values
Extremely difficult	21.95%	9.00%	0.051
Somewhat difficult	34.15%	26.00%	0.411
Neither easy nor difficult	9.76%	40.00%	0.0003
Somewhat easy	29.27%	21.00%	0.381
Extremely easy	4.88%	4.00%	1.000
Length of re-introduction phase			
< 1 month	37.50%	35.42%	0.846
1-2 months	17.50%	22.92%	0.646
3-4 months	20.00%	16.67%	0.630
5-6 months	7.50%	5.21%	0.693
> 6 months	17.50%	19.79%	0.816
Patients currently in the personalization/maintenance phase			
Yes	62%	46%	0.136
No	38%	54%	0.136
Number of high FODMAP foods patients in personalization phase are avoiding			
<3 food items	4.00%	6.10%	1.000
4-5 food items	21.00%	26.50%	0.774
6-10 food items	37.50%	30.60%	0.602
>10 food items	37.50%	36.70%	1.000

### Qualitative data results

The research team identified 5 overarching themes from the focus groups. These included: IBS symptom relief with the LFD, difficulty with restriction and reintroduction phases of the LFD, anxiety about food with the LFD, practical and emotional barriers to the LFD, and desire for simpler alternatives to the traditional approach to the LFD.

#### 1 IBS symptom relief with LFD

The participants in both focus groups reported that despite the LFD not being a perfect solution, the biggest positive of the diet was the efficacy and relief of IBS symptoms.

“…it did seem to help me” [S9G1]

Other participants concurred with this sentiment stating:

“…So then I did try it-like an earnest attempt … and I noticed really good results really fast” [S2G2]

“…And so, I was on it for about a year and was fairly successful in doing a number of things” [S4G1]

#### 2 Difficulty with restriction and reintroduction phases of LFD

While the efficacy of LFD was the most popular positive sentiment among both focus groups, the limited and restrictive nature of the diet was a common challenge.

“…the elimination was very difficult because the foods that you could eat were so limited.” [S4G2]

“…And just how much you’re limited in terms of the types of foods that you should not eat is obviously quite limiting as well.” [S3G2]

“…I didn’t like having to reintroduce things. That whole part of it was very tough … it was just a very long process.” [S6G1].

“…I think because my doctor didn’t tell me much about the reintroduction that sounded a little intimidating.” [S2G2]

#### 3 Anxiety about food with LFD

Another common theme many participants mentioned was their attitude and experiences toward food in general with regards to their IBS symptoms. Many reported a fear of food, as well as anxiety when it came to eating.

“…it’s always that question. OK, so if [sic] instead of two grapes, if I have 4. Will four kill me? You know. Yeah. It’s that kind of a thing.” [S7G1]

“…I kind of struggled with my relationship with food and being like afraid of food” [S4G2]

“…And then when you are eliminating food, then you’re going to bring it back. There is a fear factor there, you know. That’s partly why I’ve never retested. I just eat it on occasion, but I’ve never specifically, done the retest for the fodmap that I did because I I’m afraid to, to be really honest.” [S4G2]

#### 4 Practical and emotional barriers to LFD

Difficult terminology and unfamiliarity with suggested foods:

“…the accessibility of understanding the sort of scientific names versus what that actually breaks down to for the layperson and how to understand” [S3G2]

“…Yeah, the terminology is a little bit hard to grasp for the layperson” [S2G2]

“…It was just very complicated for me. The suggestions were a lot of foods that I was not familiar with.” [S9G1]

Challenges with eating outside the home:

“…Eating out. I would say going somewhere either. To a restaurant, to a friend’s home. Eating at a conference when it’s a fixed diet and you don’t have control. You know you have to bring your own things.” [S7G1]

“…socially, it’s difficult and people think people in my life think I’m overdoing it. You know a little too obsessed with it?” [S2G2]

“… I’m very conscious of that, and my children seeing me eating differently and things when we sit around having family meals.” [S8G1]

Burden of time required with grocery shopping and seeking low FODMAP recipes:

“…It wasn’t so much the food as it was the stress of I’ve got to find that in the grocery store. And how do I cook it?” [S9G1]

“…So I think the hardest thing to cook for my wife was to find really good recipes that were fodmap recipes” [S4G1]

“…My kids are young, and I’m very busy, so it’s meant that mom had to take a lot of time for herself and that’s not usually the way it is.” [S8G1]

#### 5 Desire for simpler alternatives to LFD

“…If anything is there, is there any way to make it simpler?” [S9G1]

“…Yeah, simplified version will be nice for sure.” [S8G1]

While others reported that a faster version of the diet, or a tired approach to the diet would be something they would be interested in.

“…That it would be faster. That you could do it faster. Like maybe like an allergy test. You know where you just get a little thing under your skin and it will tell you if you react to that fodmap” [S4G2]

“…Yeah, maybe there could be a, you know, a tiered approach where you know. You do like a first run … maybe if that doesn’t all work. Then maybe you could go a little deeper and because I assume simple might not work for everybody” [S2G2]

## Discussion (key results)

This study sought to better understand what challenges patients faced in the different phases of LFD and attitudes patient had toward LFD. Most survey respondents reported perceived improvement in their IBS symptoms with the LFD. However, we found that in exchange for the clinical benefits, the LFD presents significant practical challenges. Some of the challenges that were highlighted in survey data and/or qualitative data included the restriction phase being moderately or extremely restrictive, difficult to follow, and presenting social and lifestyle challenges. Participants also found the reintroduction phase difficult to navigate, while the majority were hoping for a simpler alternative overall for the LFD.

The LFD is currently recommended as a 3-step process, and GI societies consistently recommend implementing this under the guidance of an expert dietitian (17–20). However, in this real-world study, we found that 70% of participants implement the LFD without the help of a dietitian/nutritionist. This number is similar to another study performed at a tertiary care center, where only 30% saw a dietitian for low FODMAP diet guidance (14). In this real-world survey, we found that almost one in five respondents were unaware of the reintroduction phase, and only 45% were currently following the personalization phase. This is much lower than other studies from tertiary care settings, which have reported 67% of participants in the personalization phase (21). More consistent with previous studies, we found that compared to those participants who had not worked with a dietitian/nutritionist, those who had worked with a dietitian/nutritionist tended to be more likely to implement the reintroduction and personalization phases of the LFD (14). Given that one challenge of implementing LFD appeared to be the availability of dietitians/nutritionists, increasing the number of dietitians or making high-quality online resources more widely available could be seen as possible solutions for helping to guide patients.

Another observation from our study was that participants often spent more than the recommended time in each of the LFD phases. Guidelines recommend that the restriction phase should last 4–8 weeks (22); however, only 40% of participants reported this to be the case. We found that one-fifth of our participants continued the restriction phase beyond 6 months, substantially increasing the risks of micronutrient deficiencies (which have been reported with even 4–6 weeks of this phase), dysbiosis, and disordered eating (23). Similarly, expert consensus recommends the reintroduction phase to be 6–10 weeks long (22), however one-fifth of participants took >6 months to finish this phase. We found that working with a dietitian/nutritionist reduced the risk of following the restriction phase beyond 6 months, further supporting the benefits of implementing this complex diet with the help of a dietitian/nutritionist.

Some of the biggest takeaways from the qualitative group sessions were that many participants had improvement in symptoms when on LFD. However, they struggled with the terminology of the diet and had difficulty finding adequate food options/recipes. Moreover, anxiety about not doing the restriction phase correctly and the fear of being judged by others were common experiences with which many participants struggled. They also reported anxiety regarding symptom relapse as a barrier to the reintroduction process. In general, participants found the whole process time-consuming and socially isolating.

## Discussion (limitations)

Our study had several limitations. First, there is a possibility of selection bias which could have affected participant responses. To address this, a balanced Likert scale was utilized for response choices. Secondly, participants recruited via the IFFGD mailing list likely represent more highly motivated and health literate individuals. This self-selection in addition to the aspect of retrospective recall poses a risk for introducing bias. Third, our diagnosis of IBS was clinician supported and the study team in this study did not confirm IBS diagnosis with individual patients using Rome criteria (24, 25).

## Discussion (strengths)

Despite these limitations, we have several strengths. This is the largest study to date reporting patients’ experience with the LFD in a community setting. Additionally, we captured experience with all three phases, which is often lacking in other studies. Finally, our focus groups and qualitative data add further depth to our understanding of participant experience with this complex diet.

## Conclusions

We found that an overwhelming majority of participants found the LFD to be difficult, cumbersome, and restrictive. Despite recommendations from GI societies, only a minority of respondents with IBS worked with a dietitian/nutritionist while implementing the LFD, and more than half did not complete all three phases of the LFD. In focus groups, participants commonly reported food-related fear and anxiety during the restriction phase, as well as concern about symptom recurrence during the reintroduction phase. Furthermore, many participants lacked access to a dietitian/nutritionist who could guide them through the 3 phases of the LFD, leading to high rates of over-restriction and early discontinuation before reaching the personalization phase. Given the limited availability of dietitians, particularly in rural areas, many participants turned to online resources to implement the diet independently. Notably, 83% of participants expressed interest in a simplified version of the LFD, which underscores the need for more accessible and patient-centered alternatives that improves adherence and food-related quality-of-life.

### Practice implications

More counseling and education need to be done informing patients of the re-introduction and personalization/maintenance phases of the LFD. Patients may need additional counseling as well regarding how long they should be in the restriction/elimination phase. While they may be reluctant to move out of this phase due to anxieties surrounding symptom flairs, this anxiety should be acknowledged and addressed so they can more appropriately move on to the other phases of the diet. Additionally, a significant portion of patients are receiving their LFD information online or in the form of an app, and dietitians and physicians should meet patients where they are at, providing a more direct and simplified evidence-based guide that patients can access online.

## Data Availability

The original contributions presented in the study are included in the article/**Supplementary Material**. Further inquiries can be directed to the corresponding author.
