# Supplementary material for: Irritable bowel syndrome in the community: a cross-sectional study looking at patient experiences and challenges with the low FODMAP diet
Source: Front Gastroenterol (Lausanne). 2026 Oct 23;5:1930300. doi: 10.3389/fgstr.2026.1930300 (PMC13739530; doi:10.3389/fgstr.2026.1930300)
Supplement: Supplementary file 1 [file Table1.docx]

**Supplemental Table 1. Demographic characteristics of the IFFGD mailing list**

| **Participant Characteristic** | **Category** | **Percentage (%)** |
| --- | --- | --- |
| **Gender** | Male | 20 |
|  | Female | 80 |
| **Household Income** | < $25,000 | 27.4 |
|  | $25,000-34,999 | 10 |
|  | $35,000-49,999 | 9.6 |
|  | $50,000-74,999 | 15.3 |
|  | $75,000-99,999 | 10.8 |
|  | $100,000-124,999 | 9.9 |
|  | $125,000-149,999 | 5.1 |
|  | >$150,000 | 11.7 |
| **Race/Ethnicity** | White | 81.9 |
|  | Asian | 6.9 |
|  | Hispanic/Latino | 5.7 |
|  | Black/African American | 4.1 |
|  | Middle Eastern/North African | 0.5 |
|  | Hawaiian/Pacific Islander | 0.5 |
|  | Other | 4.8 |
| **Educational Status** | Less than high school | 3.8 |
|  | High school graduate | 11.3 |
|  | Trade/Technical | 4.6 |
|  | Some College | 13.9 |
|  | Associate degree | 11.0 |
|  | Bachelor’s degree | 26.8 |
|  | Advanced degree | 28.6 |
| **Military Status** | Active duty | 1.5 |
|  | Retired | 3.8 |
|  | None | 94.7 |

**Supplemental Table 2: Survey Questions Administered to Members of the IFFGD Mailing List Discussing Experience with LFD and IBS Symptoms**

| **Questions Number** | **Question** | **Answer Response Choices** |
| --- | --- | --- |
| **1** | Do you have irritable bowel syndrome ? | Yes, No |
| **2** | Who diagnosed you with IBS ? | Open response |
| **3** | Have you ever tried a low FODMAP diet to help with your IBS symptoms ? | Yes, No |
| **4** | Why not ? | The diet was too difficult to follow, the diet was too restrictive to follow, the diet was too expensive to follow, other |
| **5** | Overall, how easy or difficult was the restriction/elimination phase of the low FODMAP diet to follow? | Extremely easy, somewhat difficult, neither easy nor difficult, somewhat easy, extremely easy |
| **6** | Did you find the restriction/elimination phase of the low FODMAP diet to be restrictive/liberal in terms of what you could eat ? | Extremely restrictive, moderately restrictive, somewhat restrictive, neither restrictive nor liberal, somewhat liberal, moderately liberal |
| **7** | What was your opinion on the cost of following the restriction/elimination phase of the low FODMAP diet compared to what you would normally eat? | Extremely expensive to follow, moderately expensive to follow, somewhat expensive to follow, neither cheaper nor more expensive to follow, extremely cheaper to follow |
| **8** | Who helped you to navigate the restriction/elimination phase of the low FODMAP diet? (Choose more than one if applicable) | My doctor provided me with counseling on the diet, I worked with a nutritionist/dietician, I found resources online, I used Monash or other app, I mostly did this on my own, other |
| **9** | How long were you on the restriction/elimination phase of the low FODMAP diet? | 8 weeks or less, 8-12 weeks, 13-16 weeks, 17-20 weeks, 20 weeks or more |
| **10** | How satisfied were you with the restriction/elimination phase of the low FODMAP diet ? | Extremely dissatisfied, somewhat dissatisfied, neither satisfied nor dissatisfied, somewhat satisfied, extremely satisfied |
| **11** | Did your symptoms improve with the restriction/elimination phase of the low FODMAP diet ? | Yes, No |
| **12** | Would you be interested in a simplified version of the low FODMAP diet ? | Yes, No |
| **13** | Who helped you with the FODMAP re-introduction phase ? (Choose more than one if applicable) | My doctor provided me with counseling for the re-introduction phase, I worked with a nutritionist for the re-introduction phase, I found materials online for the re-introduction phase, I was not aware of the FODMAP re-introduction phase, Other |
| **14** | How easy or difficult did you find the re-introduction phase of the low FODMAP ? | Extremely difficult, somewhat difficult, neither easy nor difficult, somewhat easy, extremely easy |
| **15** | How long did it take you to complete the re-introduction phase ? | 1 month or less, 1-2 months, 3-4 months, 5-6 months, >6 months |
| **16** | Are you currently on the personalization/maintenance phase of the low FODMAP diet? | Yes, No |
| **17** | How many high FODMAP items do you currently avoid while being on the personalization/maintenance phase of the low FODMAP diet? | 3 items or less, 4-5 food items, 6-10 food items, >10 food items |
